# The Socio-Demographic Characteristics of Patients Diagnosed with Prostate Cancer Treated in South Africa’s Only Rural Central Hospital in 2020: A Cross-Sectional Study Protocol

**DOI:** 10.3390/healthcare14020221

**Published:** 2026-01-16

**Authors:** Xolelwa Ntlongweni, Sibusiso C. Nomatshila, Wezile W. Chitha, Sikhumbuzo A. Mabunda

**Affiliations:** 1 School of Public Health, Walter Sisulu University, Mthatha 5117, South Africa; snomatshila@wsu.ac.za (S.C.N.); wchitha@wsu.ac.za (W.W.C.); smabunda@wsu.ac.za (S.A.M.); 2 WSU Society and Health Research Institute, Walter Sisulu University, Mthatha 5117, South Africa; 3 WSU Institute for Clinical Governance & Healthcare Administration, East London 5247, South Africa; 4 WSU Global Centre for Human Resources for Health Intelligence, Walter Sisulu University, East London 5247, South Africa; 5 School of Population Health, University of New South Wales, Sydney, NSW 2052, Australia; 6 George Institute for Global Health, University of New South Wales, Sydney, NSW 2052, Australia

**Keywords:** prostate cancer, epidemiology, secondary data, rural health

## Abstract

**Background**: Prostate cancer remains a significant public health burden globally, particularly in sub-Saharan Africa, where rising incidence rates are compounded by limited screening, late-stage diagnosis and disparities in healthcare access. In South Africa, the Eastern Cape Province reports high prostate cancer prevalence, with many patients presenting at advanced stages. Understanding the epidemiological profile of affected individuals is critical for developing targeted health strategies. **Objectives**: This sub-study aims to describe the epidemiological characteristics of patients diagnosed with prostate cancer, using secondary data from Nelson Mandela Academic Hospital (NMAH), focusing on patients seen between March 2020 and November 2021. **Methods**: A quantitative cross-sectional study design is employed. De-identified secondary data extracted from clinical records of male patients diagnosed with prostate cancer and managed at NMAH during the study period. Variables include demographic information, clinical characteristics, health service utilization indicators. **Analysis**: Data will be captured and coded in Microsoft excel 2013 (Microsoft corporation, Seattle, WA, USA). The data will then be exported to STATA 18 for analyses. Descriptive statistics will be used to summarize the data. Inferential analyses such as logistic regression and chi-square tests will be used to explore associations between variables and treatment outcomes. The study provides insights into the demographic and clinical profiles of prostate cancer patients in a high-burden setting. It is anticipated that findings will highlight the age distribution, stage at diagnosis, and treatment patterns among patients diagnosed with prostate cancer. This will inform future prevention and intervention strategies in the Eastern Cape Province. **Conclusions**: By mapping out the epidemiological patterns of prostate cancer in the Eastern Cape through this sub-study, the research contributes to evidence-based planning and resource allocation, ultimately supporting efforts to reduce prostate cancer morbidity and mortality in rural South Africa.

## 1. Introduction

Prostate cancer is one of the leading cancers affecting men globally and has become an increasing public health concern in low- and middle-income countries (LMICs), including those in sub-Saharan Africa. It is the second most frequently diagnosed cancer in men, with over 1.4 million new cases reported worldwide in 2020 [1]. More than 375,000 died as a result of the disease [2]. The burden is not equally distributed; the incidence is highest in high-income countries due to established screening practices, and mortality rates are disproportionately high in low- and middle-income countries (LMICs), including those in sub-Saharan Africa [1,3]. In South Africa, prostate cancer is the most common cancer among men, with a prevalence rate five times higher than in northern America, reflecting both a high disease burden and growing diagnostic capacity [4]. It has become the leading cancer diagnosed among men, overtaking lung cancer in incidence in recent years [5]. The age-standardized incidence rate has steadily increased, and projections suggest that this trend will continue due to population aging and lifestyle changes. While improved awareness and diagnostic technologies have contributed to increased detection, especially in urban areas, disparities remain in rural regions where diagnostic delays, poor health literacy, and cultural beliefs often result in late presentations [6,7].

Although South Africa has a better diagnostic infrastructure compared to many African countries, disparities in access to cancer care persist, especially in rural provinces such as the Eastern Cape. Lusikisiki, a rural area located within the Eastern Cape’s OR Tambo District, recorded an incidence of 53.1 per 100,000, almost double the global average of 29.3 per 100,000, which suggests that improved national infrastructure does not necessarily translate into equitable access for all populations [8]. Despite the high burden, prostate cancer survivors in rural South African settings often present at advanced stages of the disease, partly due to poor health-seeking behaviors, their beliefs and perceptions, limited awareness, and over-reliance on traditional medicine [6,9].

Numerous studies have highlighted challenges in prostate cancer management in rural South Africa. Among these are systemic factors, including poor referral systems, inadequate infrastructure, a lack of urologists and oncologists, and limited access to screening services [10]. The Nelson Mandela Academic Hospital (NMAH) in Mthatha is the referral facility for cancer patients across five of the eight Eastern Cape health districts. However, detailed epidemiological data on prostate cancer patients managed at this facility remain limited. Secondary data from hospital records present an important opportunity to understand the socio-demographic and clinical profiles of prostate cancer survivors in the rural context [11].

The study aims to comprehensively describe the epidemiological characteristics of prostate cancer patients who attended NMAH between March 2020 and November 2021. Through a retrospective analysis of patient records, it seeks to detail the age distribution, stage at diagnosis, treatment modalities employed, as well as examine healthcare utilization patterns among patients diagnosed with prostate cancer, including follow-up care and resource accessibility, to inform clinical practice and healthcare planning. The term “patients” refers to individuals diagnosed with prostate cancer who received care at NMAH between March 2020 and November 2021, regardless of treatment status and survival outcome at the time of abstraction.

## 2. Materials and Methods

### 2.1. Study Design

A quantitative cross-sectional study focusing on the epidemiology of prostate cancer will be conducted among patients diagnosed with prostate cancer. This study will utilize secondary data from patients seen at Nelson Mandela Academic Hospital between March 2020 and November 2021. The cross-sectional design is suitable because it allows for the analysis of data at a specific point in time, providing a snapshot of the distribution of epidemiological factors such as age, stage at diagnosis, and treatment patterns among patients diagnosed with prostate cancer. This approach facilitates the identification of associations and healthcare utilization trends without the need for long-term follow-up, making it efficient and practical for using existing records. This is a descriptive study with analytical components. Confounders will be adjusted during the analysis stage through stratification and the comparison of rates across different groups with different age structures or other characteristics.

### 2.2. Study Setting

The study site is the Nelson Mandela Academic Hospital, a quaternary referral hospital situated in Mthatha, within the OR Tambo District municipality of the Eastern Cape Province, South Africa. NMAH is the referral hospital for several level 1 (district) hospitals and level 2 (regional) hospitals. NMAH provides specialist care services including oncology, urology, internal medicine, and surgery [12].

NMAH plays a critical role in the referral chain for cancer management in the districts it serves, particularly for urological malignancies such as prostate cancer. However, like many hospitals in rural South Africa, it is constrained by limited diagnostic and treatment resources, and patient presentations often occur at advanced disease stages [6,13]. The hospital uses a combination of electronic and paper-based medical record systems. The electronic data are captured using the hospital’s Health Information System (HIS), which stores patient demographics, diagnostic results, and treatment summaries. Complementary paper-based records are maintained for clinical notes, consent forms and pathology reports that are not yet digitized. To ensure consistency, clerks from the oncology units as well as oncology nurses extract information from both the sources following the hospital’s internal Data Management Standard Operating procedure (SOP), which outlines processes for data verification, cross referencing and secure storage. Discrepancies between paper and electronic sources are resolved through joint review by the oncology unit data manager and the clinical team before the data can be included in the study dataset. For this study, secondary data is extracted from archived files and digital records from the oncology and urology departments. These data provide valuable insights into the demographic and clinical profiles of prostate cancer survivors treated at the facility, which will help to fill a significant gap in the current rural cancer epidemiology literature.

### 2.3. Population and Sampling

Convenience sampling is used to select patients attending the Outpatient Oncology clinic in NMAH. These are patients who have been diagnosed with prostate cancer and are receiving treatment between March 2020 and November 2021. To obtain data, the records of 527 patients are reviewed, and socio-demographic data are extracted from the files. This represents a real-world patient profile managed within a resource-constrained academic hospital.

#### 2.3.1. Inclusion Criteria

Male patients with a confirmed diagnosis of prostate cancer. Patients who received treatment and/or follow-up care for prostate cancer at Nelson Mandela Academic Hospital within the study period (March 2020 to November 2021).

#### 2.3.2. Exclusion Criteria

Records with incomplete or missing essential clinical information that would compromise the accuracy or completeness of the data analysis.

#### 2.3.3. Handling of Missing Data

Missing data will be handled during the analysis stage, where they will be analyzed based on the data available for each variable. Records will not be excluded solely based on missingness. Missing variables will be managed using suitable statistical techniques to minimize bias and maintain validity.

### 2.4. Data Collection

The instrument for this study is a structured data extraction tool (S1) see Supplementary Materials, specifically designed to systematically collect and analyze secondary data from medical records of male patients diagnosed with prostate cancer and treated at the Nelson Mandela Academic Hospital’s Oncology or Urology departments between March 2020 and November 2021. It captures key variables including patient demographics (age, ethnicity), clinical information (cancer type, stage at diagnosis, treatment modalities, and clinical outcomes), as well as health service utilization metrics (number of hospital visits, types of interventions received, and follow-up care details). The development of this instrument was informed by a thorough review of the hospital’s patient record system, ensuring all selected variables are relevant and comprehensive for evaluating the clinical progression and treatment outcomes of prostate cancer patients during the specified period. Additionally, data fields were standardized and clearly defined to maintain consistency and improve comparability across all patient records reviewed.

### 2.5. Data Analysis

Data will be captured and coded in Microsoft Excel 2013 (Microsoft Corporation, Seattle, USA) and subsequently exported to STATA 18 for analysis. Descriptive statistics will summarize the data using frequency tables, graphs, means (with standard deviation and range), or medians (with interquartile range), depending on whether the numerical variables follow a normal distribution. The Shapiro–Wilk test will be used to test for the normality of distribution of numerical variables.

For numerical variables:

The *t*-test or the Wilcoxon rank-sum test will be used to compare the mean or median ages of patients from two referral facilities. These tests help determine whether there are statistically significant differences in patient ages across groups, such as those diagnosed early versus late, thereby highlighting potential disparities in diagnosis timing.

ANOVA or Kruskal–Wallis will be used to test continuous outcomes across multiple groups, such as varying cancer stages or treatment types, multiple districts, sub-districts, or health facilities. This enables the identification of significant differences in age or other continuous variables, helping to understand how these factors relate to disease progression or treatment decisions.

For categorical variables:

Logistic Regression will be applied to identify factors influencing the likelihood of a successful treatment outcome, adjusting for multiple covariates. This analysis helps determine which patient or clinical characteristics are predictive of a better prognosis, guiding targeted interventions. Model diagnostics will be undertaken using linearity in the logit, collinearity, outliers, and goodness-of-fit. The 95% Confidence Interval (95%CI) will be used to determine the precision of estimates. Analyses will further be clustered by referral health facility or sub-district where appropriate.

The chi-square test will assess the associations between categorical variables, such as treatment modality and patient outcomes. It helps establish whether observed differences in outcomes are statistically meaningful, supporting evidence-based clinical decision-making.

Together, these statistical methods will enable a comprehensive evaluation of patterns, relationships, and predictors within the patient data, providing insights into epidemiological and clinical factors that affect prostate cancer management and outcomes.

### 2.6. Validity and Reliability

Validity has been ensured by selecting a sample that represents the study’s objectives and the broader patient population of males that is treated at NMAH for prostate cancer. The instruments will be evaluated for the validity of the content by consulting experts in prostate cancer and public health to ensure that the tools comprehensively cover all relevant aspects of prostate cancer. Reliability will be addressed by adopting rigorous data handling procedures and thorough cleaning of the data.

### 2.7. Ethical Aspects

This study has received ethical approval from the Human Research Ethics Committee of the Faculty of Medicine and Health Sciences at Walter Sisulu University (Reference Number: 014/2025). Additionally, access approval is granted by the Eastern Cape Provincial Research Committee (Reference Number: EC_202503_009) and the Nelson Mandela Academic Hospital. As this is a secondary data analysis, only de-identified patient records are used to ensure confidentiality and privacy. No direct contact is made with study participants, and no personally identifiable information is collected or reported.

## 3. Limitations

This study relies on secondary data extracted from medical records; the quality and completeness of the data are dependent on the accuracy of clinical documentation and record-keeping practices at the hospital. Missing or incomplete entries may limit the robustness of certain variables. Another limitation is that the study design does not allow for control over the selection of variables beyond what is available in the records; for instance, the absence of data on environmental and occupational exposures, particularly to heavy metals such as cadmium, which recent evidence has identified as potential contributors to prostate cancer risk [14]. Variables such as patients’ environmental conditions, lifestyle factors, or occupational history were not routinely captured. As a result, the study is unable to account for those contextual risk determinants or evaluate their possible influence on the onset of the disease, progression, or treatment outcomes. This is a limitation that should be considered when conducting future research to gain a more comprehensive understanding of prostate cancer determinants in a region such as the Eastern Cape. Despite these limitations, the systematic approach to data extraction and standardization of variables will help to minimize inconsistencies and provide valuable insights into the demographic and clinical characteristics of male cancer patients in the rural central hospital context.

## 4. Dissemination

The findings of this study will be disseminated through a comprehensive, multi-level approach to maximize accessibility and impact across academic, clinical, and community audiences. At the academic level, results will be submitted for publication in peer-reviewed, open-access journals such as Healthcare (MDPI) to ensure wide availability to researchers and clinicians globally. Additionally, the findings will be presented at national and international conferences focused on oncology, health systems, and public health, facilitating scholarly discussion and knowledge exchange.

Clinically, key findings will be communicated directly to stakeholders at Nelson Mandela Academic Hospital and the Eastern Cape Department of Health through formal reports and presentations. This dissemination will support evidence-based decision-making and inform local cancer care policies and resource allocation. To engage the community, simplified summaries and infographics will be developed in both English and isiXhosa, the local language, ensuring accessibility for prostate cancer survivors, their families, and patient advocacy groups. These materials will be shared in community forums, support groups, and through local health outreach programs across the Eastern Cape. Interactive sessions may also be organized to discuss the findings and gather feedback, reinforcing community involvement and empowerment. Through this multifaceted dissemination strategy, the study aims to foster informed clinical practices, policy improvements, and community awareness around prostate cancer care.

## Data Availability

The data sharing is not applicable to this article. This is a study protocol and does not include datasets or analyzed results. Upon completion of the study, the data will be made available in accordance with the ethical approvals and policies of the Walter Sisulu University ethics committee.

## Supplementary Materials

The following supporting information can be downloaded at: https://www.mdpi.com/article/10.3390/healthcare14020221/s1.

## References

[1] Gandglia G., Leni R., Bray F., Fleshner N., Freeland S.J., Kibel A., Stattin P., van Poppel H., La Vecchia C. (2021). Epidemiology and prevention of Prostate Cancer. Eur. Urol. Oncol..

[2] Sung H., Ferlay J., Siegel R.L., Laversanne M., Soerjomataram I., Jemal A., Bray F. (2021). Global Cancer Statistics 2020: GLOBACON Estimates of Incidence and Mortality Worldwide for 36 Cancers in 185 Countries. CA A Cancer J. Clin..

[3] Rawla P. (2019). Epidemiology of prostate cancer. World J. Oncol..

[4] Hamdi Y., Abdeljaoued-Tej I., Zatchi A.A., Abdelhal S., Boubaker S., Brown J.S., Benkahla A. (2021). Cancer in Africa: The Untold Story. Front. Oncol..

[5] National Cancer Registry (2019). Cancer in South Africa: Annual Report 2019.

[6] Cassim N., Ahmad A., Wadee R., Rebbeck T.T., Glencross D.K., George J.A. (2021). Prostate cancer age-standardised incidence increases between 2006 and 2016 in Gauteng Province, South Africa: A laboratory data-based analysis. South Afr. Med. J..

[7] Ramaliba T.T., Tshilidzi M., Mphaphulu E. (2022). Trends and patterns of prostate cancer in South Africa: 2010–2017. Afr. J. Urol..

[8] Eastern Cape Department of Health (2020). Eastern Cape Cancer Registry Report: 2013–2017.

[9] Miller D.B., Markt S.C., Nguyen C.T., Coleman O.C. (2022). Prostate Cancer screening and young black men: Can early communication avoid later health disparities?. J. Cancer Educ..

[10] Hoffman A., Amiel G.E. (2023). The impact of PSMA PET/CT on modern prostate cancer management and decision making—The Urological perspective. Cancers.

[11] De-Graft Aikins A., Unwin N., Agyemang C., Allotey P., Campbell C., Arhinful D. (2010). Tackling Africa’s chronic disease burden: From the local to the global. Glob. Health.

[12] Eastern Cape Department of Health (2021). Annual Performance Plan 2021/2022.

[13] Jacobs R., Matlakala F.K., Mulaudzi F.M. (2020). Cultural beliefs of African men: Implications for prostate cancer screening. Curations.

[14] Firmani G., Chiavarini M., Dolcini J., Quarta S., D’Errico M.M., Barbadoro P. (2024). The Association Between Cadmium Exposure and Prostate Cancer: An Updated Systematic Review and Meta-Analysis. Int. J. Environ. Res. Public Health.

