# Supplementary figures and images for: The Socio-Demographic Characteristics of Patients Diagnosed with Prostate Cancer Treated in South Africa’s Only Rural Central Hospital in 2020: A Cross-Sectional Study Protocol
Source: Healthcare (Basel). 2026 Jan 16;14(2):221. doi: 10.3390/healthcare14020221 (PMC12841281; doi:10.3390/healthcare14020221)

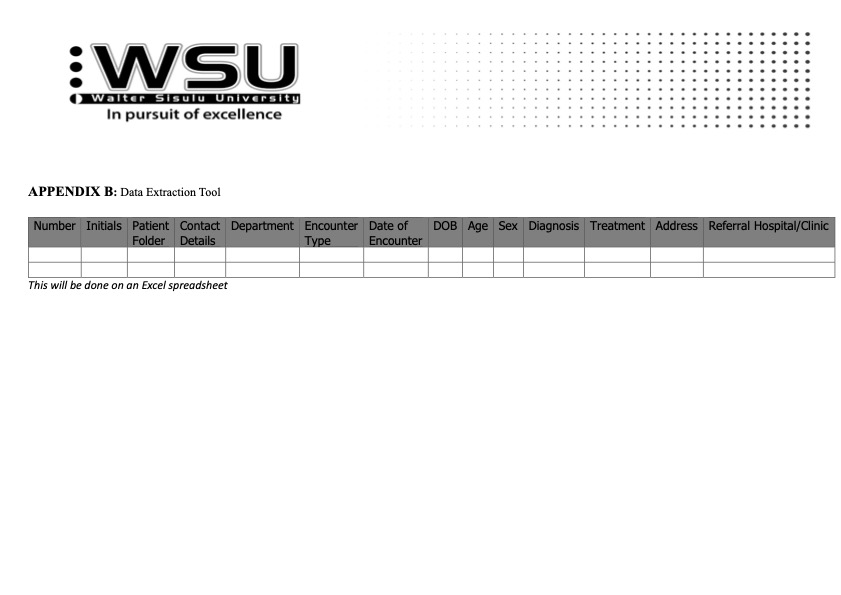

Supplement: Supplementary file 1 [file healthcare-14-00221-s001.zip › healthcare-3937552-supplementary.jpg]
